# ‘Click, I Guess I’m Done’: Applicants’ and Assessors’ Experiences Transitioning to a Virtual Multiple Mini Interview Format

**DOI:** 10.5334/pme.1035

**Published:** 2023-12-29

**Authors:** Zoe Abraham, Carolyn Melro, Sarah Burm

**Affiliations:** 1 Dalhousie University, Halifax, Nova Scotia, Canada; 2 Department of Continuing Professional Development and Division of Medical Education at Dalhousie University, Halifax, Nova Scotia, Canada

## Abstract

**Introduction:**

During the COVID-19 pandemic, medical schools were forced to suspend in-person interviews and transition to a virtual Multiple Mini Interview (vMMI) format. MMIs typically comprise multiple short assessments overseen by assessors, with the aim of measuring a wide range of non-cognitive competencies. The adaptation to vMMI required medical schools to make swift changes to their MMI structure and delivery. In this paper, we focus on two specific groups greatly impacted by the decision to transition to vMMIs: medical school applicants and MMI assessors.

**Methods:**

We conducted an interpretive qualitative study to explore medical school applicants’ and assessors’ experiences transitioning to an asynchronous vMMI format. Ten assessors and five medical students from one Canadian medical school participated in semi-structured interviews. Data was analyzed using a thematic analysis framework.

**Results:**

Both applicants and assessors shared a mutual feeling of longing and nostalgia for an interview experience that, due to the pandemic, was understandably adapted. The most obvious forms of loss experienced – albeit in different ways – were: 1) human connection and 2) missed opportunity. Applicants and assessors described several factors that amplified their grief/loss response. These were: 1) resource availability, 2) technological concerns, and 3) the virtual interview environment.

**Discussion:**

While virtual interviewing has obvious advantages, we cannot overlook that asynchronous vMMIs do not lend themselves to the same caliber of interaction and camaraderie as experienced in in-person interviews. We outline several recommendations medical schools can implement to enhance the vMMI experience for applicants and assessors.

## Introduction

The Multiple Mini Interview (MMI) prevails as the preferred interview method for student selection in medical education [1]. First introduced in 2004, the MMI is a highly structured interview format resembling the Objective Structured Clinical Examination (OSCE) [2]. While the implementation of MMIs varies in different settings and locations, they typically consist of multiple short assessments, each overseen by a volunteer assessor. Each interview station focuses on a different question or scenario aimed at measuring a wide range of non-cognitive competencies important to the practice of medicine, such as teamwork, communication, and decision-making [2,3]. Several studies reveal the MMI to be a fair and effective admission tool, with moderate to high reliability [2,3,4,5,6]. Furthermore, the predictive validity of the MMI is exemplified in the work undertaken by Eva et al. and Kim et al., showing the MMI to be an excellent predictor of subsequent OSCE performance, clerkship performance, and licensing examination outcomes [3,5,6,7,8,9,10]. Compared to traditional unstructured interview formats, the MMI has been favoured across stakeholder groups (i.e., applicants, assessors, admission committee members) [1]. For these compelling reasons, the MMI has been widely adopted by medical schools worldwide.

When the COVID-19 pandemic hit, medical schools were forced to suspend in-person interviews and transition to a virtual MMI (vMMI) format. This adaptation required medical schools to make swift changes to their MMI structure, delivery, and scoring system. As a result, medical school applicants and MMI assessors were thrust into largely unfamiliar interview contexts, as reliance on web-based technologies skyrocketed in response to the uncertainty and growing number of COVID-19 cases [11,12,13,14]. Inevitably, a change of this scale incites logistical, technological, and equity considerations demanding closer examination. Several studies have been conducted evaluating the feasibility and validity of the vMMI format for medical school admissions processes [15,16,17,18,19]. However, this literature largely focuses on innovation and technological advancement [16,20,21,22,23,24,25] and the effectiveness of vMMIs compared to the in-person format [18,26,27,28]. Absent from this work is any fulsome acknowledgement of the human experience shifting to the vMMI format and, more specifically, what individuals lose when ‘business as usual’ admission processes pivot online. We address this gap by focusing on two specific groups greatly impacted by the decision to move MMIs online: medical school applicants and MMI assessors. Understanding these groups’ perspectives on transitioning from one interview modality to another may offer meaningful insights into the vMMI format as more than just a tool that enabled medical schools to get through the pandemic, but rather as an opportunity to better understand the vMMI as a complex interaction mediated by the affordances and constraints of people, spaces, technologies, and objects. In this paper, we draw on sociomateriality [29,30,31,32,33] as a method for understanding the various material and social elements assembling to produce the vMMI experience described by applicants and assessors. In this study, ‘material elements’ encompass a spectrum of things, ranging from bodies and videoconferencing equipment to physical space, as well as less apparent but essential items for participating in vMMIs, such as electricity and internet infrastructure. By ‘social’, we are referring to the cultural discourses and specific rules and conventions influencing how individuals behave in various social situations, such as an admissions interview. Those who embrace a sociomaterial perspective share the belief that individuals are never alone in pursuing a specific course of action. In this view, both social and material forces exert agency, contributing to what may seem like ‘normal’ or ‘natural’ outcomes in education [29,30,31,32,33]. In this paper, we sensitize readers to how a seemingly inconsequential transition to a vMMI format can produce certain ‘effects’, subsequently altering the medical school admission process for applicants and assessors. Our research question was as follows: What were medical school applicants’ and assessors’ experiences transitioning from in-person to vMMIs?

## Methods

We conducted an interpretive qualitative study [34,35] to explore medical school applicants’ and assessors’ experiences facing the forced change to vMMIs during the pandemic. Our interest was in learning more about how applicants and assessors fared during this potentially stressful transition and what they perceived to be the affordances and drawbacks of relying on video conference technology to inform medical school admission decisions. Ethics approval to conduct this inquiry was received from the Health Sciences Research Ethics Board at Dalhousie University (Reference #: 2021–5603).

In the 2020/2021 application cycle, Dalhousie University officially transitioned to vMMI format. This shift involved applicants participating in an asynchronous vMMI consisting of five interview stations, each approximately five to seven minutes in length. Assessors reviewed and scored roughly 40 to 50 applicants’ video responses within a two-week time period. Previously, MMIs were conducted in-person and consisted of 10 interview stations and two rest stations, each lasting approximately eight minutes. Assessors scored applicants in real time throughout the interview day.

### Sample and data collection

Seeking a diverse pool of participants, we invited medical school applicants, current medical students, and MMI assessors to participate in semi-structured interviews. We felt these groups were best positioned to speak about the utility of both interview modalities as well as some of the unforeseen challenges that result from computer-mediated interaction. To be eligible for inclusion, participants must have previously engaged in both the in-person and vMMI at Dalhousie University. Potential participants were identified and recruited through relevant social media groups (e.g., ‘Ask a Med Student’ Facebook group) and through distribution of an email invitation sent by the Dalhousie Undergraduate Medical Education Admission Office. Enrolled participants were also asked to share information about our study with peers who they felt fit our inclusion criteria (snowball sampling). In total, 10 assessors and five medical students from the class of 2025 agreed to participate.

Data collection occurred from January 2021 to January 2022. Due to public health restrictions at the time, research interviews were conducted virtually by ZA and CM using MS Teams. Interview questions were open-ended to yield descriptive data. There were targeted questions specifically for medical school students (e.g., ‘Tell me about how your experience with the in-person MMI compared to your experience with the vMMI.’), MMI assessors (e.g., ‘Tell me about your training and preparation for the virtual MMI.’), as well as questions directed to both groups (e.g., ‘If you participate in another MMI, what format would you prefer and why?’). Interviews were approximately 40–80 minutes in length, digitally recorded, and transcribed verbatim by ZA or a professional transcriptionist.

### Data analysis

Interview transcripts served as the data source for analysis. We utilized Braun & Clarke’s thematic analysis framework to categorize important themes identified across the data [36]. Data analysis occurred inductively, meaning that the research team developed themes/categories from the data rather than imposing previously theoretically derived concepts [37]. Preliminary data analysis was undertaken by the first author (ZA), who familiarized herself with the transcribed data in relation to the research aims and existing body of literature through recurrent reading. Throughout this phase, the research team (ZA, CM, SB) met regularly to define and discuss initial codes relevant to developing themes. Then, ZA collated the most relevant codes to our research focus (e.g., structure, environment, human interaction, accessibility) and applied these to the entire data set. At this stage, the research team continued to meet; however, our analytic work shifted toward further refining those themes that spurred valuable insights in relation to our topic of inquiry. Nvivo, a qualitative data analysis software, was used to assist with the organization and coding of data. This collaborative process, which additionally included reflexive notetaking [38], continued until we decided based on our interpretative authority [39] and pragmatic considerations that we had generated a sufficient understanding of both the people and processes enmeshed within the phenomenon of interest. Our research team is thoughtfully composed of individuals with pertinent experience and methodological expertise that complement the objectives of this study. All team members are active participants in and contributors to the field of medical education. ZA is a current medical student with a keen interest in medical school admission processes. CM holds a PhD in Health and is an active contributor to the health professions education field. SB holds a PhD in Education Studies and leads an active program of research that falls broadly under the umbrella of social accountability.

## Results

In the wake of the pandemic, the transition from an in-person to a virtual asynchronous MMI format evoked a wide range of emotions amongst participants, from uncertainty and stress to gratitude and relief. However, it was the sense of loss described and felt by both applicants and assessors that captured our attention. Our analysis revealed both groups sharing a mutual feeling of longing and nostalgia for an interview experience that, due to the pandemic, was understandably adapted and lost. The most obvious forms of loss both groups were experiencing – albeit in different ways – were: 1) loss of human connection and 2) missed opportunity. Both participant groups also discussed several factors that amplified their grief/loss response. The quotes featured below are identified by participant type and number. For example, A1 indicates a quote attributed to Assessor #1 and M1 indicates a quote attributed to Medical School Applicant #1.

### Loss of human connection

As a result of changes to the structure and delivery of the MMI, applicants and assessors missed ‘the human interaction’ (M1) and ‘community engagement’ (M2) that the in-person format enabled, indicating that the vMMI was really isolating and lonely’ (A1). When probed further, applicants discussed the absence of in-the-moment support and encouragement from upper-year medical students and faculty who typically would be present on interview day. Assessors shared this perception, stating that one of the benefits of the in-person interview was that applicants who ‘come from out of town…have a chance to meet other people who are applying’ (A2).

As video interviewing was a relatively new experience for many, both groups expressed concern that the absence of face-to-face interaction would leave applicants ‘more disadvantaged’ (A3). For assessors, they lost the ability to participate in helpful conversations and ask questions of one another throughout the interview day: ‘[In-person, you had the] opportunity to speak to people with the same station. And I think this is really important. [You would] get together with a group of people doing [your] station and talk about… what you’re looking for.’ (A2). For applicants, the perceived impersonal nature of the virtual asynchronous format was considered a potential handicap to the evaluation of their suitability for medicine, resulting in a ‘less individualized experience’ (M3), and making it ‘harder to stand out and show your true self’ (M2). In comparison to their in-person experience, it seemed the virtual interview was unsatisfying for some applicants, in large part because of the lack of real-time connection: ‘my best stations were the ones where I had the best conversations…I was talking to a person. And I’m sure that’s relevant to medicine as well’ (M1). Furthermore, applicants realized how much they relied on assessors’ non-verbal behaviours to appraise their performance at each interview station: ‘Having someone physically [present], I felt like I was better able to gauge how I did at each of the stations. When you’re sitting in front of your computer you have no idea how you’re chopping up.’ (M4).

### Missed opportunity

With fewer interview questions and less time allotted per question in the virtual format, both participant groups felt applicants had limited opportunity to elaborate on the experiences and attributes that distinguished them from other applicants. One applicant recalled thinking to themselves ‘wow, this is really short. I feel like they’re not getting to know people very well.’ (M2). Some applicants expressed frustration for not having ‘a chance to fully complete their answer’ (M5) while others described feeling rushed, ‘like you would only get half a thought out.’ (M4). Assessors were of the same opinion, stating that: ‘[In-person], if one or two assessments were not fair or well thought out or accurate, they would be observed over the spread amongst 10 questions. But with five questions [in the virtual format], it… puts more eggs in one basket.’ (A4). Additionally, in-person, assessors could ‘probe where they needed to’ (A5), if ‘someone was going off topic or they missed the key point’ (A1). Assessors felt that in-person:

You have the opportunity to see how [applicants] think on their feet… You get a truer view of who somebody really is. With the video interviews, more of them compared to the live sessions were what I would call very staged, you know, exactly as you would expect answers, with a real lack of spontaneity and authenticity to them (A5).

Assessors noted that the asynchronous virtual format limited their ability to explore applicant responses in depth. Applicants concurred, leaving many dispirited and doubting their interview performance.

### Factors that Amplified a Grief/Loss Response

Although participants cited clear affordances to asynchronous vMMIs, namely the scheduling flexibility and reduction of logistical complications associated with travel, new challenges arose, leaving many participants feeling uncomfortable and even second-guessing the supposed benefits of virtual interviewing. The most significant factors that amplified a sense of loss were: 1) resource availability, 2) technological concerns, and 3) the virtual interview environment.

### Lack of resource availability

Applicants found their interview anxiety exacerbated with the transition from in-person to vMMIs because of their unfamiliarity with this new form of interviewing and the minimal resources available to them. Applicants described preparing for the in-person interview by watching YouTube videos, accessing question banks, and speaking to friends with previous interviewing experience. However, when preparing for the virtual interview, applicants described feeling overwhelmed and ‘apprehensive about what the interviews would look like’ (M1). They found that ‘so much of the [MMI] history is irrelevant. [You can’t go] back on forums or talk to previous med school students. There’s nothing they can say because they haven’t experienced this. No one has’ (M1). They agreed that ‘the more practice and prep material, the better, so you can understand what you’re going into’ (M3). Applicants found that their preparation methods ‘had to change’ (M4) significantly, with a large focus on recording and rewatching themselves, as well as ‘learning how to use the [new] technology’ (M2).

### Technological concerns

Applicants also expressed anxiety about the reliability of their technology and Wi-Fi connection: ‘My computer is from 2010. It’s still working, but what if the internet cuts out? What if the computer itself stops working?’ (M1). Another applicant who lived in a rural community expressed unease about their internet connectivity, electing to make alternative arrangements in preparation for their vMMI:

We’re in the middle of nowhere, so I had to come [into the city] for both my interviews because our internet sucked, and I didn’t really know anyone with great internet… If I had not had the opportunity to… utilize my friend’s internet access, I don’t think I would have been as successful (M5).

Assessors agreed, stating that internet is indeed a barrier for some applicants, amplifying stress levels during an already stressful time: ‘[At school], I see my tutor… with crappy Wi-Fi, freezing with an odd expression…and I just imagine that happening to these poor, already super stressed, interviewees’ (A6).

### The virtual interview environment

Applicants affectionately recalled their in-person MMI experiences, describing themselves being surrounded by highly motivated individuals in a professional hospital setting. They described how this environment ‘changes your demeanor’ (M4) and makes you feel ‘mentally sharp’ (M3). Not surprisingly, transitioning to the virtual interview environment was challenging for some. As one applicant articulated: ‘You’re in your bedroom. You’re in a place that is not normally… a place of business, a place of professionalism, [or] a place of focus.’ (M1). Applicants missed the sense of camaraderie and competitive excitement previously felt in-person, sharing that ‘the environment and setting was really crucial’ (M4).

Further concern was expressed regarding space and logistical limitations. Both participant groups reported difficulty in finding a ‘clean and quiet’ (A1) space to effectively focus on the virtual interview. This task was made increasingly difficult by social distancing regulations designed to prevent the spread of COVID-19. Applicants acknowledged that reliance on videoconferencing meant the private spaces of their home (e.g., home offices, bedrooms) would be public, raising concerns about whether their video background would lead to being unfairly evaluated. One applicant expressed, ‘you’re concerned about the lighting… the technology… noises, background, anything like that’ (M1).

Finally, in the virtual environment, applicants described an anti-climatic feeling once their interview concluded: ‘Click, I guess I’m done. There’s no de-stress debrief afterwards.’ (M1). Another applicant echoed this, stating: ‘It didn’t really feel like it was over… I felt kind of happy but there were no people to hang out with; nothing to do. It was just like oh, okay, now I’m going to have lunch. [Laughs]. I didn’t feel the same kind of excitement… it didn’t really feel like I had done an interview.’ (M2). Assessors missed the opportunity for more dialogue as well, describing a sense of unease and discontentment following the virtual interview process: ‘I felt I made much more of a contribution in the in-person situation. I felt that I was much more in control of assessing in a way that I could stand behind and be proud of… I did not feel that confidence with the online ones.’ (A4). Both participant groups felt that the lack of resource availability, technological concerns, and factors introduced by the virtual environment resulted in an overall sense of unease and disappointment.

## Discussion

In this study, we interviewed medical school applicants and assessors to explore their transitional experiences from the in-person MMI to an asynchronous vMMI format. The results of our study illuminate a sense of loss and, to a certain degree, some ambivalence toward the transition to vMMI during the pandemic. Applicants expressed concern regarding their ability to self-assess their personal ‘fit’ with the medical school. In addition, they expressed feeling disadvantaged by the virtual format, citing new sources of potential bias which left many doubting their interview performance. Meanwhile, assessors felt less certain about their evaluative role, lacking confidence in their ability to fairly evaluate an applicant’s suitability for a career in medicine based on an asynchronous recorded video. While both participant groups understood why in-person MMIs were unfeasible during COVID-19, they expressed a common sentiment: asynchronous vMMIs do not lend themselves to the same caliber of interaction and camaraderie that can be experienced in-person.

We can appreciate why the widespread use of vMMIs became the “new normal” for many medical schools amid the COVID-19 pandemic. As noted earlier, the literature concerning the overall feasibility and effectiveness of administering MMIs virtually is rapidly growing. There is no doubt that virtual interviewing has clear benefits, such as improved efficiency, cost savings, and greater scheduling flexibility [14,16,17,18,23,24,25,26,28,40,41]. However, our study participants offer a contrasting perspective worth heeding. Both applicants and assessors appeared unconvinced that asynchronous vMMIs could effectively compensate for the structured and carefully timed interview stations they were intimately familiar with. We know from other published work the importance of directly observing social interactions, non-verbal cues, and applicants’ interpersonal communication skills in real-time [11,14,20,42,43]. We are also cognizant that applicants struggle with the perceived social pressure to perform in a virtual setting [28]. Lastly, we cannot deny the increased cognitive load of virtually evaluating social interactions that traditionally have always occurred in person [44].

Drawing on sociomateriality, we can see how the vMMI is far from being a neutral activity. Rather, the resulting sociomaterial assemblage of the vMMI is dynamic and forceful enough to actively influence and, consequently, produce an array of behaviours and outcomes, whether intended or unintended [29,30,31,32,33]. While the pivot to virtual interviewing removed barriers that were once imposed by distance, we cannot ignore that this near replication of the admissions interview presents unique, potentially unforeseen challenges related to formality, intent, and accessibility for applicants and assessors alike.

Pickering describes this interplay of social and material elements as the “mangle of practice,” where individuals and materials are mutually “engaged in the play of resistance and accommodation” [45]. From a sociomaterial perspective, it becomes apparent that addressing the challenges faced by applicants and assessors during vMMIs is not about assigning blame or rectifying individual actions. Instead, it involves making visible the intricate network of human and non-human actors that assemble during the interview process. Our intent here is not to dissuade medical schools from utilizing virtual platforms for facilitating MMIs. Instead, we aim to remind them of what they risk compromising if the implementation lacks systematic monitoring and critical reflection.

Based on our findings, we offer a few practical recommendations for medical schools to consider that may enhance the vMMI experience. First, regarding the modality and delivery of the vMMI, it is critical to maintain connections between the medical school, the applicants, and the assessors, as they may be participating from drastically different environments. Schools will need to identify new ways to foster connection in a virtual setting. For applicants, medical schools may consider fostering social connections via virtual tours and get-togethers, greater social media engagement (e.g., day in the life videos from current students), virtual presentations highlighting the strengths of their undergraduate medical education program and information concerning the city where the medical school is located (e.g., housing affordability, child care and transit options etc.), as well as live breakout rooms to freely mingle and debrief post-interview [21,46,47,48]. Similarly, for assessors, medical schools may consider live breakout rooms, discussion boards, or regularly scheduled meetings throughout the academic year to create a space for assessors to debrief and provide feedback following the assessment period.

In contrast to the in-person format, the vMMI does not provide a standardized environment for all stakeholders. Therefore, we secondly urge medical schools to think critically about the new forms of bias (e.g., background noise and appearance, lighting, audio and camera quality) that can unwittingly influence ratings of applicants’ performance when private spaces serve as the backdrop in a high-stakes selection setting. We recommend medical schools consult with campus centres that have expertise in technology, diversity, inclusion, and accessibility [47] and implement mandatory anti-bias training that is specific to the vMMI format [46]. In collaboration with these centres, we further recommend that schools provide applicants with a checklist that outlines optimal attire and appearance, background setup, technology checks, and additional tips for interview day [22,23].

Finally, as video interviewing strategies continue to advance, medical schools will need to broaden the types of research questions they ask with greater focus on the social dynamics and material elements at play [29,30,31,32,33]. We cannot ignore the effect that the interview medium has on both applicants’ and assessors’ participation in, and reactions to, the selection process.

### Strengths and Limitations

Our study began in 2020 at the height of the pandemic, and more specifically, just after the first round of vMMIs took place at our study site. Thus, we were limited by our population as only one cohort of applicants and assessors had participated in both an in-person and asynchronous vMMI. We acknowledge the number of pandemic-driven innovations that have materialized over the last few years to optimize the vMMI process. We recognize there are clear benefits to conducting admission interviews virtually and are not suggesting vMMIs be abandoned. Rather, our discussion centres on providing readers with a closer look at why computer-mediated interviews remains a fascinating and inconclusive debate in medical education. In this paper, we focus on the student selection processes in undergraduate medical education; however, we see our findings transferrable to postgraduate medical education (e.g., medical residency matching services) and other health professions education programs. In this study, sociomateriality served as a high-level framework for highlighting the diversity of actors influencing the implementation of vMMIs. We recognize, however, that there are more specific sociomaterial approaches such as actor-network theory (ANT), or cultural historical activity theory (CHAT) that can be utilized to critically examine the ways in which materials influence human activity. See work by Fenwick and Dahlgren [49], and Frambach, Driessen, and Van der Vleuten [50] for related theoretical approaches.

In conclusion, our study investigated the experiences of medical school applicants and assessors who participated in both the in-person and asynchronous vMMI. Overall, both participant groups felt that the asynchronous vMMI was lacking the human interaction and camaraderie they previously experienced in-person. There is no doubt that virtual interviewing has obvious advantages, but delving further into applicants’ and assessors’ perspectives offers new insight into what can be lost when medical schools shift from an in-person to virtual interview environment. In addition to outlining the implications of our findings, we have provided several recommendations that medical schools can utilize or adapt to enhance the vMMI experience for applicants and assessors. We hope this research will encourage medical schools to reflect on their use of digital technology and critically consider the unintended consequences transitioning to a virtual environment can reveal.
